# External and internal intensity profiles of older male and female participants during a walking football game

**DOI:** 10.1371/journal.pone.0318286

**Published:** 2025-06-30

**Authors:** Júlio A. Costa, Catarina Pereira, Ana Barbosa, André Seabra, João Brito, Ana Pinto, Catarina Martins, Rafaela Moreira, Bruno Gonçalves

**Affiliations:** 1 Portugal Football School, Portuguese Football Federation, FPF, Oeiras, Portugal; 2 Departamento de Desporto e Saúde, Escola de Saúde e Desenvolvimento Humano, Universidade de Évora, Évora, Portugal; 3 Comprehensive Health Research Centre (CHRC), Universidade de Évora, Évora, Portugal; 4 EPIUnit ITR, Institute of Public Health of the University Porto, University of Porto, Porto, Portugal; SPRINT - Sport Physical Activity and Health Research & Innovation Center, PORTUGAL

## Abstract

**Background:**

Walking football (WF) can be used as an impactful activity for healthy aging and decrease the high levels of sedentary behavior among older adults. This study examines the external and internal intensity profiles of male and female participants during a WF tournament, addressing a gap in research on game demands and induced load.

**Methods:**

The study involved 176 players aged 50 + participating in a 40-min, 5v5 WF tournament with unlimited substitutions. External intensity profiles (total and categorized distances) were measured using Global Positioning System (GPS), while heart rate (HR) monitors assessed internal intensity profiles, including absolute HR and intensity zones based on %HRmax.

**Results:**

The proportion of male participants (n = 123; 70.3%) was higher than females (n = 52; 29.7%), p < .001. They were similar in age (61.6 ± 8.6 and 60.8 ± 6.9, respectively). Males covered a higher distance per minute than females, with sex showing a moderate effect (63.3 ± 10.7 m/min vs. 54.7 ± 15.8 m/min; p < .001; Cohen’s *d*_*unbiased*_ = 0.69 [0.36; 1.03]), especially in fast walking (41.7 ± 12.2 m/min vs. 32.6 ± 16.7 m/min; p < .001; Cohen’s *d*_*unbiased*_ = 0.66 [0.33; 1.00]). Males played more time than females (22:26 ± 09:47 min:ss vs. 15:41 ± 07:46 min:ss; p < .001), with moderate effect (Cohen’s *d*_*unbiased*_ = 0.73 [0.40; 1.06]). However, no differences between sexes were identified in the intensity load variables, such that the female average %HRmax was 80 ± 11% and the male was 82 ± 8% during the practice.

**Conclusions:**

Overall, while males generally exhibit higher external intensity profiles in WF, both sexes experience similar internal intensity profiles, highlighting WF’s potential as a scalable, health-promoting intervention for aging populations.

## Introduction

Literature reports a substantial growth in the body of evidence on the multiple health and well-being benefits of different types, intensities, and durations of physical activity, as well as the negative impact of sedentary behavior on health [1]. Physical activity in older ages is a known protective factor for mental health and prevention and management of noncommunicable diseases, such as cardiovascular disease, type 2 diabetes, or cancer [1,2] Physical activity also improves functional fitness, mobility and ability, therefore contributing to older adults independence and well-being [3].

Several international organizations, such as the World Health Organization (WHO) [1], the American College of Sports Medicine (ACSM) [4], and the Centers for Disease Control and Prevention (CDC) [5], recommend that adults, including older adults, engage in at least 150 minutes of moderate-intensity or 75 minutes of vigorous-intensity aerobic physical activity per week, or an equivalent combination of both, spread throughout the week. For older adults, these guidelines also emphasize the importance of multicomponent physical activity that includes aerobic, balance, and strength components on at least three days per week to maintain functional capacity and reduce fall risk [6].

Despite well-established physical activity guidelines, adherence among older adults remains suboptimal. Research indicates only modest improvements over time, with many older individuals continuing to exhibit high levels of inactivity that increase with age [7,8]. Recognizing this, the WHO’s Global Action Plan on Physical Activity 2018–2030 explicitly calls for “appropriately tailored programs and services aimed at increasing physical activity and reducing sedentary behavior…” (Action 3.4) [9]. In this context, walking has been widely endorsed as a practical and accessible activity for older adults, with proven benefits for cardiovascular health, functional mobility, mood, and overall quality of life [10].

Walking Football (WF), a modified format of soccer for individuals aged 50 years and older, emphasizes inclusivity and safety by prohibiting running and physical contact [10]. Its growing popularity is tied to the recognition of physical activity as a vital determinant of health for aging populations [11]. WF offers a low-impact, scalable activity that can elicit light-to-vigorous intensity exercise with a relatively low risk of injury [10]. Evidence highlights its potential to enhance physical fitness and foster social connectedness in older adults [10,12], yet the physiological demands of WF remain underexplored, especially concerning sex-based differences. Specifically, while external intensity metrics (e.g., distance covered, movement speeds) provide insight into the mechanical demands of the activity, intensity responses such as heart rate (HR) zones offer a view into cardiovascular strain [10,12]. However, limited research exists comparing these metrics between male and female participants, which this study aims to address through a detailed analysis of sex-specific external and internal intensity responses during WF game. In fact, while sex differences in physical activity responses have been documented in other exercise modalities for older adults, such as resistance training and aerobic interventions [13], these differences are rarely examined in game-based or sport-specific formats like WF.

Therefore, this study investigates sex-specific differences in WF by analyzing external and internal intensity profiles during a game. It was hypothesized that males would exhibit higher external intensity profiles (e.g., distance and speed) than females, while intensity (%HRmax) would remain similar due to the consistent moderate-intensity demands of WF, based on known sex-based differences in aerobic capacity, muscle mass, and gait mechanics that typically promotes greater physical output in men [14].

## Methods

### Study design

This study utilized a cross-sectional observational analytical design to assess participants’ external and internal intensity profiles in WF games during a national WF game held in Oeiras, Portugal, in 2023.

### Participants

Participants were eligible for the study if they were aged ≥ 50 years and provided a valid medical fitness certificate issued by a licensed medical doctor confirming their suitability for sports participation. The recruitment process, conducted by the project manager via email and in person, took place between June 1^st^ and July 31^st^ 2023 and targeted each football association in Portugal. No dropouts or excluded participants were found.

Participants provided written informed consent prior to participation. Ethical approval was obtained from the Portugal Football School Ethics Committee (Protocol number: 21/CEPFS/2023), and the study was conducted in compliance with the Declaration of Helsinki.

### Procedures

WF games were played under standardized rules tailored for participants over 50 years of age: no running with or without the ball; maximum of three touches on the ball by player, no physical contact, including slide tackles [11]. Besides these rules, it was defined that the ball must always be played below the players’ average waist height [15]. Games were held on a 40x20 meters and lasted 40 minutes, divided into two 20-minute halves with a 10-minute interval. Teams consisted of five outfield players, and substitutions (n = 5 players) were unlimited. Each team played four games. It is important to mention that, regarding familiarization, all participants were already accustomed to the walking football modality, as they regularly engaged in this activity through their local clubs prior to the tournament.

Games were played in an outdoor natural grass. Data were collected across all 12 games of the tournament by trained researchers with a Human Kinetic Sciences background. Each researcher performed always the same evaluation task.

### External intensity profiles

External intensity profiles were captured using 10 Hz Global Positioning System (GPS) devices (STATSports Apex, Northern Ireland). The devices were turned on 10 min before the WF games and placed on the participants who wore a customized and specific neoprene vest in the midline between the scapulae at the level of the seventh cervical vertebra (C7). Distances were categorized by speed zones distance covered as: distance covered at speeds < 4 km/h (low-intensity activity); distance covered at speeds > 4 km/h (higher-intensity activity) [10,16]. The reliability and validity of the GPS devices were previously established in sports performance studies [17].

### Internal intensity profiles

To measure internal intensity during WF games, a GARMIN HR (Garmin Ltd., Olathe, Kansas, United States) monitoring band was used by the participants [18]. Absolute HR values were recorded and the percentage of age-estimated maximal HR (%HRmax) was calculated using the equation HRmax = 211 − 0.64 × age [19]. However, if players reached HR values during the game that exceeded this predicted maximum, those observed values were instead used as their HRmax. Average HR, % HRmax, HRpeak were registered, and the player’s HR data were categorized into intensity zones: Zone 1: < 50% HRmax; Zone 2: 50–60% HRmax; Zone 3: 60–70% HRmax; Zone 4: 70–80% HRmax; Zone 5: 80–90% HRmax; Zone 6: > 90% HRmax [10] (adapted for walking football older participants) [16].

### Statistical analysis

After preliminary inspections for distribution and assumptions, an independent sample t-test analysis was processed to identify the effect of the sex (female vs male) on the considered variables. The statistical analysis was performed using the Statistical Package for the Social Sciences software (SPSS, Inc., Chicago, IL, USA), and statistical significance was set at p < .05.

An estimation techniques approach was carried to overcome the shortcomings associated with traditional N-P null hypothesis significance testing [20,21]. The Cohen’s *d*_*unbiased*_ (*d*_*unb*_) with 95% confidence intervals (CI) as effect size (ES) (an unbiased estimate has a sampling distribution whose mean equals the population parameter being estimated) was applied to identify pairwise differences between sexes [20]. The thresholds considered were 0.2, 0.5, and 0.8 for small, medium, and large effect sizes, respectively [22].

## Results

A total of 175 participants (52 females and 123 males) were included in the study. The mean age was 61.8 ± 6.9 years for females and 60.8 ± 6.9 years for males, ranging from 50–76 years old.

All study participants played all the games in their friendly tournament bracket during the WF tournament. No adverse events were observed.

The descriptive and inferential results of the effect of sex on the independent variables are presented in Table 1.

**Table 1:** Descriptive and inferential analysis.

Variables	Female	Male	Paired *t*	*p* value	Cohen *d*_*unbiased*_ [95% CI]
Age (years)	61.6 ± 8.6	60.8 ± 6.9	−0.39	.696	−0.11 [−0.66; 0.44]
Played time (min)	15.7 ± 7.8	22.4 ± 9.8	4.41	**<.001**	0.73 [0.40; 1.06]
**Heart Rate**					
Heart Rate Peak (BPM)	161.5 ± 20.7	161.3 ± 17.8	−0.06	.955	−0.01 [−0.39; 0.37]
Maximal (%HRmax)	93.6 ± 6.4	94.7 ± 6.5	0.51	.614	0.16 [−0.46; 0.79]
Average (BPM)	140.8 ± 20	139.3 ± 18.8	−0.37	.714	−0.08 [−0.49; 0.33]
Average (%HRmax)	80.1 ± 11.0	82.0 ± 8.1	0.62	.537	0.21 [−0.46; 0.88]
% Time in Zone 1 (<50%HRmax)	2.9 ± 8.5	0.1 ± 0.6	−1.85	.072	−0.62 [−1.3; 0.06]
% Time in Zone 2 (50–60%HRmax)	4.5 ± 8.6	1.9 ± 4.5	−1.31	.198	−0.44 [−1.12; 0.23]
% Time in Zone 3 (60–70%HRmax)	11.4 ± 21.9	9.1 ± 13.8	−0.44	.665	−0.15 [−0.81; 0.52]
% Time in Zone 4 (70–80%HRmax)	18.3 ± 16.5	23.3 ± 18.3	0.83	.413	0.28 [−0.39; 0.95]
% Time in Zone 5 (80–90%HRmax)	42.5 ± 28.5	38.8 ± 21.7	−0.46	.651	−0.15 [−0.82; 0.51]
% Time in Zone 6 (>90%HRmax)	20.4 ± 28.6	26.6 ± 29.7	0.63	.534	0.21 [−0.46; 0.88]
**Distance covered (m/min)**					
Total Distance	54.7 ± 15.8	63.3 ± 10.7	4.20	**<.001**	0.69 [0.36; 1.03]
Distance < 4 km/h	22.5 ± 3.1	21.8 ± 3.0	−1.47	.142	−0.24 [−0.57; 0.08]
Distance ≥ 4 km/h	32.6 ± 16.7	41.7 ± 12.2	4.03	**<.001**	0.66 [0.33; 1.00]

**Fig 1:**
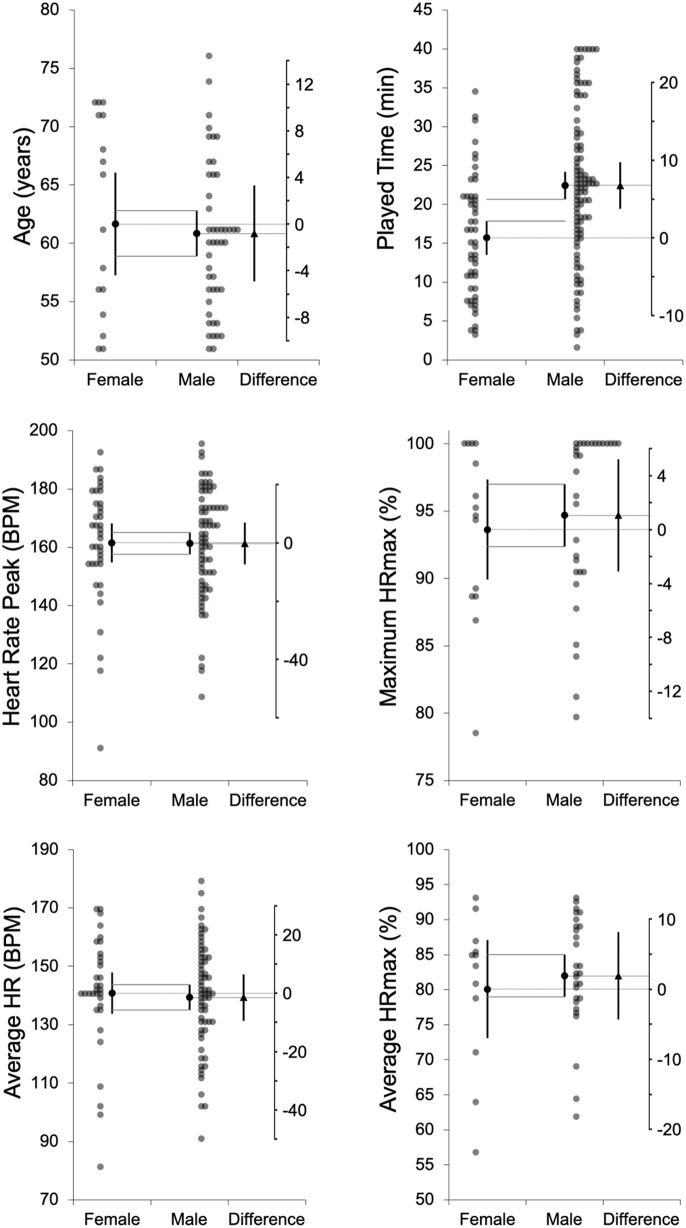
Playing time, and physiological variables. Means and 95% confidence intervals for age, playing time, and physiological variables, presented separately for females and males. The mean difference between groups, along with its 95% confidence interval, is shown on the floating difference axis on the right, aligned with the female group mean on the primary axis.

**Fig 2:**
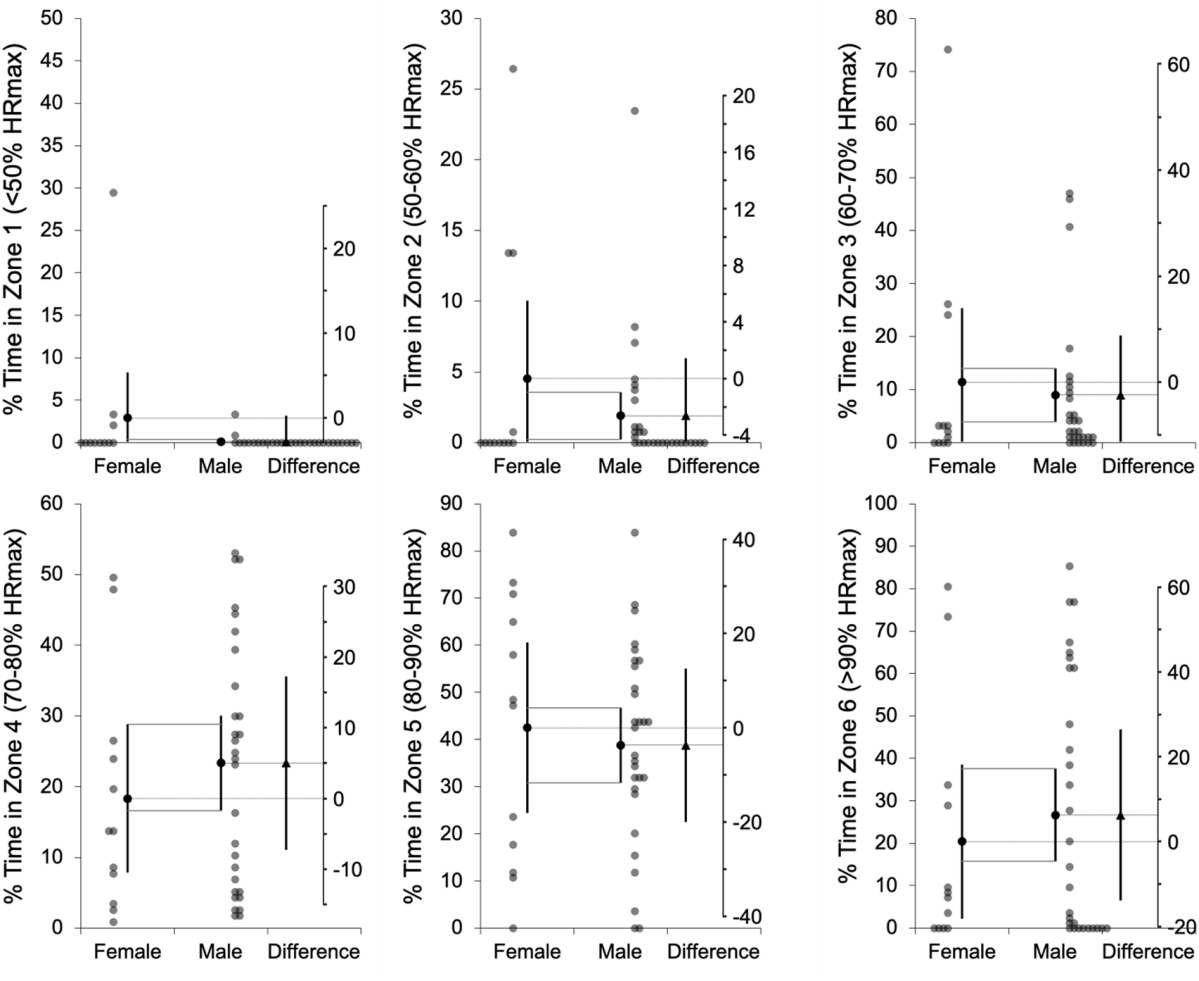
Percentage of time spent in each heart rate zone. Percentage of time spent in each heart rate zone (%HRmax) across six intensity zones (<50%, 50–60%, 60–70%, 70–80%, 80–90%, and >90% HRmax), presented separately for females and males. The mean difference between groups, with its 95% confidence interval, is displayed on the floating difference axis to the right, aligned with the female group mean on the primary axis.

**Fig 3:**
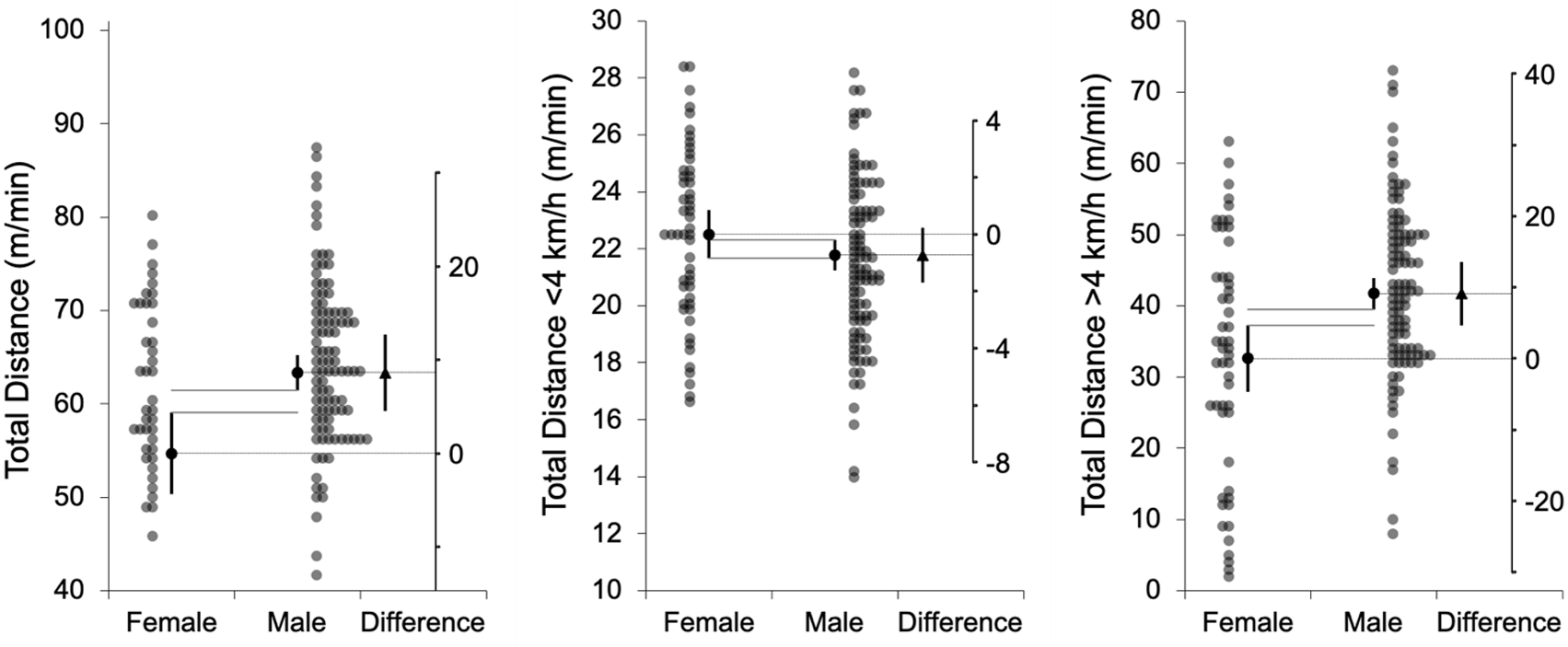
Total distances covered in different speeds. Total distance covered (m/min), and distance covered at speeds below (<4 km/h) and above (>4 km/h), presented separately for females and males. The mean difference between groups, with its 95% confidence interval, is shown on the floating difference axis to the right, aligned with the female group mean on the primary axis.

**Fig 4:**
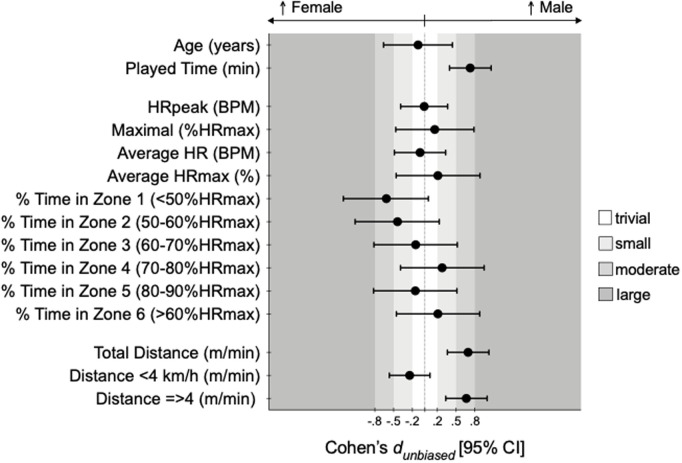
Cohen’s dunbiased differences for considered according to sex. Error bars indicate uncertainty in the true mean changes with 95% confidence intervals.

Additionally, individual differences and mean values from independent comparisons are shown in estimation plots: Figs 1–3 for age, playing time, intensity, and distance metrics. Complementarily, Cohen’s *d*_*unbiased*_ (*d*_*unb*_) with 95% confidence intervals for all comparisons are illustrated in Fig 4.

Concerning external intensity profiles, males covered significantly greater total distances per minute than females (female: 54.7 ± 15.8 m/min vs. male: 63.3 ± 10.7 m/min; t = 4.20, p < .001; *d*_*unb*_ = 0.69 [0.36; 1.03]). This difference was especially pronounced at speeds above 4 km/h (female: 32.6 ± 16.7 m/min vs. male: 41.7 ± 12.2 m/min; t = 4.03, p < .001; *d*_*unb*_ = 0.66 [0.33; 1.00]). Conversely, distances covered at speeds below 4 km/h did not differ significantly between sexes (t = −1.47, p = .142; *d*_*unb*_ = −0.24 [−0.57; 0.08]).

Males played significantly longer time compared to females (female: 15:41 ± 07:46 min:ss vs. male: 22:26 ± 09:47 min:ss; *t* = 4.41, *p* < .001; *d*_*unb*_ = 0.73 [0.40; 1.06]). Regarding internal intensity, no significant sex differences were observed in HR variables, including average BPM (*t *= −0.37, *p* = .714; *d*_*unb*_ = −0.08 [−0.49; 0.33]), maximum BPM (*t* = −0.06, p = .955; *d*_*unb*_ = −0.01 [−0.39; 0.37]), or %HRmax across all intensity zones, such that female average %HRmax was 80.1 ± 11.0% and male was 82.0 ± 8.1 during the WF games.

## Discussion

The findings of this study provide valuable insights into the physiological demands of WF (i.e., analyses the external and internal Intensity) profiles among male and female participants during a WF tournament. By examining external and internal Intensity profiles, this research highlights the potential of WF to offer moderate-to-vigorous intensity exercise, with nuanced differences based on sex.

The results revealed that male participants exhibited higher external intensity profiles compared to their female counterparts, covering greater distances at faster speeds. This aligns with established evidence that males generally have higher absolute aerobic capacity and muscle mass, contributing to greater physical outputs during exercise [14]. However, the observed differences in external intensity profiles also underscore the need for tailored programming to ensure that WF remains accessible and beneficial for all participants. For instance, males covered significantly more distance and played longer than females, likely reflecting differences in match exposure and possibly team dynamics or substitution strategies. To balance the volume and ensure equitable engagement, organizers could consider implementing more structured substitution schedules, minimum playing time parts, or rotating roles across games. Additionally, adjusting team sizes or slightly modifying pitch dimensions could help tailor the physical demands to individual fitness levels. For female participants in particular, providing longer match durations or increasing frequency of game participation may enhance total physical activity load without compromising safety. These adjustments would allow for equitable health benefits while maintaining the integrity and inclusiveness of the WF experience.

Contrary to the differences in external intensity profiles, internal intensity profiles (i.e., %HRmax) did not differ significantly between sexes. Both male and female participants reached and maintained HR within the moderate-to-vigorous intensity range, supporting the applicability of WF as a cardiovascular exercise [10,12,23]. These findings corroborate earlier studies that have identified WF as a suitable activity for improving heart health in older populations, regardless of sex [10,16]. The lack of significant differences in %HRmax further reinforces the potential of WF to serve as a scalable intervention for diverse demographic groups.

WF’s ability to elicit moderate-to-vigorous intensity exercise is particularly significant given the decline in physical activity levels often observed in aging populations [8]. By meeting recommended intensity thresholds [1,4,5], WF can contribute to improved cardiovascular health, enhanced muscular endurance, and better weight management [12].

The study’s findings also highlight WF’s potential in addressing sex-specific health concerns. For instance, females, who are more prone to osteoporosis and balance-related issues, may benefit from the sport’s low-impact movements that promote bone density and stability [24]. Conversely, males, who often face greater risks of cardiovascular events can influence WF’s aerobic benefits to mitigate such risks.

While this study offers robust insights, certain limitations warrant consideration. The cross-sectional design prevents causal inferences about the long-term health impacts of WF, and the lower representation of female participants though reflective of actual tournament demographics across Portuguese districts may limit the generalizability of sex-based findings. Nonetheless, a key strength of this study is that it is the first to evaluate both external and internal intensity metrics in a real-world WF tournament context, providing a detailed analytical profile of older male and female participants relative to international physical activity guidelines. Future research should adopt longitudinal designs to investigate the sustained health benefits of WF and recruit more diverse and representative samples to explore cultural and environmental influences on participation. Additionally, individualized adaptations to WF, such as adjusting game intensity, establishing personal thresholds, or modifying rules, could improve inclusivity and optimize outcomes for participants with different health statuses and fitness levels.

## Conclusion

Overall, although males generally played more time than females and covered a higher distance per minute, showing a higher external intensity profiles, both males and females report similar intensities ranging from moderate to vigorous exercise intensity. By addressing sex-specific demands, WF holds significant promise as a scalable intervention for aging populations. Continued research and innovation into WF is essential to fully harness its potential and extend its reach to broader populations.
